# Experimental data on impact of social norms on energy reforms and petition signing

**DOI:** 10.1016/j.dib.2021.107215

**Published:** 2021-06-11

**Authors:** Faraz Farhidi, Vahid Khiabani

**Affiliations:** a Georgia State University, Atlanta, GA, USA; b Middle Tennessee State University, Murfreesboro, TN, USA

**Keywords:** Energy reform, Social norms, Petition, Field experiment: Block design, Cross-state comparison

## Abstract

The first experimental data includes four groups; thus, four survey links have been designed for each group, which was sent via email. The approved target population is twelve thousand Georgia State University affiliates among faculty, staff, and students. We randomly picked three thousand for each group. Six thousand were randomly selected from the faculty and staff, and six thousand from the students. In the survey, we asked for generic information on individuals’ characteristics such as gender, income, US native or non-native, and occupation. We provided information on the impact of carbon emission on the environment and human lives and how we can alter that by switching energy-based fuels.

In the second and more comprehensive dataset, we reduced the groups to three, based on our power analysis on the first attempt. Instead of an across nations comparison (US vs. EU and China), we performed a state-level comparison. We also added a question about their political affiliation to control for prior beliefs. We executed the experiment—at the same time—in two different states, one Democrat and one Republican, but in the same region and with similar geographical boundaries. We selected Arizona (as a red state having a republican governor) and New Mexico (as a blue state having a Democrat governor). Arizona uses 9 percent renewable energy, and New Mexico utilizes only 8 percent. To control for subjects’ judgment about the norm, we compared the energy use from each of the selected states, with two different pioneer states using renewable, one blue and one red, within the same range percentage usage. We chose South Dakota with 38 percentage and Maine with 36 (at the time of execution). We wanted to rule out the subjects’ prior beliefs and preferences on each state they were about to compare with that design.

One can use these data to replicate the analysis of the reference paper entitled “Impact of the Social Norms on Energy Reform Petitions: Cross-State Comparisons.” Or since the collected data is random, there could be some side analysis on the control variables in the dataset (wage, education, political affiliation, age, gender, birthplace, marital status, and participating in an environmentally friendly Act).

## Specifications Table

**Table:** 

Subject	Energy economics
Specific subject area	Impact of social norms on energy reforms in an environmentally friendly context
Type of data	Tables
How data were acquired	Surveys
Data format	Raw
Parameters for data collection	For the first dataset, all the subjects were Georgia State University affiliates (student, staff, and faculty), and they have been chosen, randomly. For the second dataset, the subjects were Arizonan and New Mexican, from general population, and they have also been chosen randomly.
Description of data collection	For both datasets, surveys have been distributed via emails which included the unique link to each survey, and the surveys were set on the designated Qualtrics webpage.
Data source location	First dataset: Institution: Georgia State University City/Town/Region: Atlanta/GA Country: USA Second dataset: General population who registered on the Qualtrics database City/Town/Region: Arizona and New Mexico Country: USA
Data accessibility	With the article Instructions for accessing these data: Everyone can use this dataset.
Related research article	F. Farhidi, V. Khiabani, Impact of the Social Norms on Energy Reform Petitions: Cross-State Comparison, Energy Policy, 154, 112257 [1]

## Value of the Data

• Graduate students mainly can benefit from this data to replicate the reference paper's analysis, practice and conduct the power analysis, and perform extra tests.
• This data was collected randomly from the general population and educated population, including the primary covariates such as gender, income, education, marital status, number of children, and political affiliation. Thus, some side analyses can take advantage of the mentioned variables in the relevant topics.
• Since the pilot experiment was at the university level, which deviated from the general population in the main experiment, one can notice the impact of education and income on the environmentally friendly decision and energy reforms, and how can those biases affect the analysis.

## 1 Data Description

First excel sheet contains demographical questions that are asked in the attached survey, and whether subjects would sign the petition (in favour of energy reform) or not. And if they would sign, which of the proposed tax policy they preferred.

Second excel sheet contains demographical questions (including political affiliation) that are asked in the attached surveys, and only whether subjects would sign the petition (in favour of energy reform).

## 2 Experimental Design, Materials and Methods

We designed a survey, asking for energy reform, moving away from fossil-based energy to renewables [2]. We considered two channels in the implementation phase. First, one percent increase sales tax to subsidize fossil-based energy producers to adopt other technology; second, penalizing fossil-based energy producers with the ten percent Pigouvian carbon tax finance the producers who want to invest in renewables.

The experimental method includes four petitions. We have designed and emailed four different survey links for each group. Since the approved target population for the first dataset was twelve thousand faculty, staff, and students at Georgia State University, we randomly picked three thousand to each group, half from the faculty and staff and the other half from the students. In the survey, after asking subjects’ characteristics, such as gender, income level, US native or non-native, and occupation, we attempted to raise environmental awareness by providing information on the effect of carbon emission on the environment and human lives.

The survey contains energy utilization in the US. Around eighty percent of the US energy consumption is supplied by fossil-based energy, producing 15 billion metric tons of carbon dioxide. The forests required to sequester the produced carbon every year in the US are more than 15 times the existing forests in the US. Coal carbon emissions are 25 times more than solar PV to produce the same amount to generate electricity. This figure is more than double about natural gas. Still, around one-fifth of the total energy generated by coal because it is marginally cheaper and available, excluding the environmental damages it causes.

The US utilizes more fossil fuel than European countries, but less than China, even though China is faster to substitute renewables [3]. By providing energy information about the countries using more clean energy than the US, we initially hoped to nudge individuals to support renewable energy adaptation. In the first treatment, we added the comparison between the United States and European countries as a descriptive norm. This number is 45 percent for European countries, while it is around 80 percent for the US. The comparison was between the US and China. China uses 89 percent of fossil fuel-based resources. Since China uses more fossil-based energy, it might be helpful to verify the possible downturn effect of the social norm; in this case, people might think there is another country that is worse when concerning the environment. Participants would ask: “why should we care?” And the third is the comparison between the US, European countries, and China to verify the impact of having access to the complete information. At the end of the petition, we asked participants if they are willing to sign the petition or not. If they agreed to sign, we would follow another question, asking whether they prefer sales tax increment or carbon tax reform on fossil-based energy producers to cover the costs. While the first treatment would directly affect the household's costs, the latter indirectly increases individuals’ living costs.

We added an additional question in which the subjects would choose the carbon tax rather than the sales tax. We thought it would be vital if the subjects believe that it is not a hypothetical survey and have actual consequences. Thus, we added a paragraph in the petition that states that we plan to submit the outcome of this petition to Governor Deal. Since there was a high cost associated with signing the petition in favor of renewables, we speculated that less likely subjects signed it. This assumption gave us a powerful tool to identify the social behavior capability. Coming out of fossil fuel-based energy and using more clean energy helps restore the environment by limiting the negative externalities and can slow down climate change by reducing carbon emissions.

Based on the first dataset, we found three significant caveats. First, the analysis was under-power; second, there was an educational bias since the surveys were distributed at the university level; and third, subjects’ prior beliefs about other nations made the norms ineffective. In the second experiment, we corrected for those as follow:

Instead of having three treatment groups (four in total), we reduced it to one control group and two treatments. We collected data from the general population across two states with similar geographical boundaries. Instead of a country-level comparison, we executed a state-level comparison. We added a question on people's political affiliation to control for prior judgment. We experimented—at the same time—in Arizona (a red state) and New Mexico (a blue state). Arizona uses 9 percent renewable energy, and New Mexico utilizes 8 percent. To control subjects’ beliefs on the comparison norm, we compared the energy use from each of the states, with two other states leading in renewables, one has a Democrat governor, and the other had a Republican governor, with almost the same percentage of renewable energy use. We chose Maine with 36 percent renewable and South Dakota with 38. We did not include the finance channels in the second experiment.

We used the numbers in the first dataset to calculate the correct sample size in the second experiment. The total sample displays the number of the required respondents in both compared groups. The alpha represents the Type I error, and the power shows the one minus Type II error (beta) in this statistical test. The table below shows the power analysis among the three survey groups based on that data. We took advantage the following formula to compute the sample size (used one-way ANOVA pairwise analysis):

$$n=\left(p_{a}\left(1-p_{a}\right)+p_{b}\left(1-p_{b}\right)\right){\left(\frac{z_{1-\alpha /2\tau }+z_{1-\beta }}{p_{a}-p_{b}}\right)}^{2}$$

$$z=\frac{p_{a}-p_{b}}{\sqrt{\frac{p_{a}\left(1-p_{a}\right)}{n}+\frac{p_{b}\left(1-p_{b}\right)}{n}}}$$

Based on the above computation, the required respondents for each state were: 1116, which made the total equal to 2232.

**Table:** 

Power analysis among different groups			
Analysis	Total sample	Alpha	Power
T1 (63%) VS T2 (78%)	188	0.05	0.8
T1 (63%) VS T3 (56%)	1021	0.05	0.8
T2 (78%) VS T3 (56%)	91	0.05	0.8

## Ethics Statement

The authors want to thanks Middle Tennessee University State to fund and support the second experiment, collecting the state level data.

IRB approval has been obtained for both experiments, from Georgia State University's officials.

IRB Number: H17677 Reference Number 1: 345,083 Reference Number 2: 358,839

We hereby confirm that informed consent was obtained for experimentation with human subjects.

## CRediT Author Statement

**Faraz Farhidi:** Conceptualization, Methodology, Validation, Formal Analysis, Investigation, Writing – Original Draft, Writing – Review & Editing, Project Administration; **Vahid Khiabani:** Resources, Funding Acquisition.

## Declaration of Competing Interest

The authors declare that they have no known competing financial interests or personal relationships which have or could be perceived to have influenced the work reported in this article.

## Supplementary materials

Image, application 1

Image, application 2
