# Supplementary material for: Experimental data on impact of social norms on energy reforms and petition signing
Source: Data Brief. 2021 Jun 11;37:107215. doi: 10.1016/j.dib.2021.107215 (PMC8217675; doi:10.1016/j.dib.2021.107215)
Supplement: Supplementary file 2 [file mmc2.docx]

**Code**

//** Code has been written in STATA

//**Experiment 1

clear

cls

set more off

insheet using "C:\Users\ffarhidi1\Documents\Papers\Survey\Doc\Data1.csv"

egen nq1 = group(q1)

egen nq3 = group(q3)

egen nq5 = group(q5)

egen nq6 = group(q6)

egen nq7 = group(q7)

egen nq8 = group(q8)

egen nq9 = group(q9)

rename nq1 gender

rename q2 age

rename nq3 occupation

rename q4 income

rename nq5 education

rename nq6 birthplace

rename nq7 maritalstatus

rename nq8 kidsno

rename q92 sign

rename q10 taxplan

gen tax=taxplan-1

gen control=0

gen treat1=0

gen treat2=0

gen treat3=0

gen tax1=0

gen tax2=0

replace control=1 if group==1

replace treat1=1 if group==2

replace treat2=1 if group==3

replace treat3=1 if group==4

replace tax1=1 if tax==0

replace tax2=1 if tax==1

//**Graph

graph bar control treat1 treat2 treat3, bargap(100)

graph bar tax1 tax2, bargap(200)

//**comparison test among control and treatments

ttest control==treat1

ttest control==treat2

ttest control==treat3

ttest treat1==treat2

ttest treat1==treat3

ttest treat2==treat3

//**Main analysis

prob sign treat1 treat2 treat3, vce(robust)

estimates store reg1

prob sign treat1 treat2 treat3 age gender occupation education birthplace maritalstatus kidsno , vce(robust)

estimates store reg2

prob sign treat1 treat2 treat3 age gender occupation education income birthplace maritalstatus kidsno , vce(robust)

estimates store reg3

esttab reg1 reg2 reg3 using Main.rtf, se(5) re

//*** Tax

prob tax2 treat1 treat2 treat3 if sign==1, vce(robust)

estimates store reg1

prob tax2 treat1 treat2 treat3 age gender occupation education birthplace maritalstatus kidsno if sign==1, vce(robust)

estimates store reg2

prob tax2 treat1 treat2 treat3 age gender occupation education income birthplace maritalstatus kidsno if sign==1, vce(robust)

estimates store reg3

esttab reg1 reg2 reg3 using Tax.rtf, se(5) re

//**Robustness check oversampling of the second treatments

sample 80 iftreat2==1

prob sign treat1 treat2 treat3, vce(robust)

estimates store reg1

prob sign treat1 treat2 treat3 age gender occupation education birthplace maritalstatus kidsno , vce(robust)

estimates store reg2

prob sign treat1 treat2 treat3 age gender occupation education income birthplace maritalstatus kidsno , vce(robust)

estimates store reg3

esttab reg1 reg2 reg3 using Robustness.rtf, se(5) re

//**Experiment 2

clear

cls

set more off

insheet using "C:\Users\ffarhidi1\Documents\Papers\Survey\New\Data\Data2.csv"

egen nq1 = group(q1)

egen nq2 = group(q2)

egen nq5 = group(q5)

egen nq6 = group(q6)

egen nq7 = group(q7)

egen nq8 = group(q8)

egen nq9 = group(q9)

rename nq1 gender

rename nq2 state

rename q3 age

rename q4 inc

rename nq5 education

rename nq6 birthplace

rename nq7 maritalstatus

rename nq8 politicalview

rename nq9 sign

gen petition=sign-1

gen control=0

gen treat1=0

gen treat2=0

gen income=inc/1000

gen age2=age*age

replace control=1 if group==1

replace treat1=1 if group==2

replace treat2=1 if group==3

//**Graph

//graph bar control treat1 treat2, bargap(100)

//**Main analysis

prob petition treat1 treat2 if state==1, vce(robust)

estimates store reg1

prob petition treat1 treat2 if state==2, vce(robust)

estimates store reg2

prob petition treat1 treat2, vce(robust)

estimates store reg3

esttab reg1 reg2 reg3 using Main1.rtf, se(3) re

prob petition treat1 treat2 age education birthplace maritalstatus politicalview gender if state==1, vce(robust)

estimates store reg4

prob petition treat1 treat2 age education birthplace maritalstatus politicalview gender if state==2, vce(robust)

estimates store reg5

prob petition treat1 treat2 age education birthplace maritalstatus politicalview gender, vce(robust)

estimates store reg6

esttab reg4 reg5 reg6 using Main2.rtf, se(3) re

prob petition treat1 treat2 age education birthplace maritalstatus politicalview if gender==1, vce(robust)

estimates store reg7

prob petition treat1 treat2 age education birthplace maritalstatus politicalview if gender==2, vce(robust)

estimates store reg8

esttab reg7 reg8 using femalemale.rtf, se(3) re

//** to interpret marginal

margin, dydx(*)

//**Side analysis

reg politicalview education income birthplace maritalstatus gender state, vce(robust)

estimates store reg9

reg income education birthplace maritalstatus gender state age age2, vce(robust)

estimates store reg10

esttab reg9 reg10 using side.rtf, se(3) r(2) re
